# Lung-Targeted Delivery of Cepharanthine by an Erythrocyte-Anchoring Strategy for the Treatment of Acute Lung Injury

**DOI:** 10.3390/pharmaceutics14091820

**Published:** 2022-08-29

**Authors:** Jinpeng Zheng, Caihong Lu, Meiyan Yang, Jiejie Sun, Jinbang Zhang, Yuanyuan Meng, Yuli Wang, Zhiping Li, Yang Yang, Wei Gong, Chunsheng Gao

**Affiliations:** 1 State Key Laboratory of Toxicology and Medical Countermeasures, Beijing Institute of Pharmacology and Toxicology, Beijing 100850, China; 2 School of Pharmacy, Guangxi Medical University, Nanning 530021, China; 3 College of Pharmacy, Henan University, Kaifeng 475000, China

**Keywords:** Cepharanthine, nanoparticles, erythrocyte-anchoring strategy, acute lung injury, lung-targeted delivery

## Abstract

As one of the most frequent complications of critical illness, acute lung injury (ALI) carries a high risk of clinical morbidity and mortality. Cepharanthine (CPA) has significant anti-inflammatory activity, however, due to poor water solubility, low bioavailability, and short half-life, it fails to provide effective clinical management measures. Here, we explored the flexibility of an erythrocyte-anchoring strategy using CPA-encapsulated chitosan-coating nanoparticles (CPA-CNPs) anchored onto circulating erythrocytes for the treatment of ALI. CPA-CNPs adhered to erythrocytes successfully (E-CPA-CNPs) and exhibited high erythrocyte adhesion efficiency (>80%). Limited toxicity and favorable biocompatibility enabled further application of E-CPA-CNPs. Next, the reticuloendothelial system evasion features were analyzed in RAW264.7 macrophages and Sprague-Dawley rats. Compared with bare CPA-CNPs, erythrocyte-anchored CNPs significantly decreased cellular uptake in immune cells and prolonged circulation time in vivo. Notably, the erythrocyte-anchoring strategy enabled CNPs to be delivered and accumulated in the lungs (up to 6-fold). In the ALI mouse model, E-CPA-CNPs attenuated the progression of ALI by inhibiting inflammatory responses. Overall, our results demonstrate the outstanding advantages of erythrocyte-anchored CPA-CNPs in improving the pharmacokinetics and bioavailability of CPA, which offers great promise for a lung-targeted drug delivery system for the effective treatment of ALI.

## 1. Introduction

The lung is an internal surface of the body in mammals that is constantly exposed to external environmental stimuli including allergens, pathogens, chemical weapons, pollution, and viral and bacterial infections, all of which often result in activation of cell death, oncogenesis, and inflammatory [1]. For example, the COVID-19 pandemic is caused by a coronavirus, the severe acute respiratory syndrome coronavirus 2 (SARS-CoV-2) [2]. It is a rising viral infection that has infected 551,746,184 people, induced a cytokine storm followed by acute lung injury/acute respiratory distress syndrome (ALI/ARDS), and caused 6,356,176 deaths as of 30 June 2022 [3,4,5]. ALI/ARDS, a clinical syndrome characterized by an uncontrolled inflammatory response, affects more than 200,000 people in the United States each year, confers substantial mortality as high as 26–58%, and has no specific therapeutic options [6,7].

Cepharanthine (CPA, C_37_H_38_N_5_O_6_), a bis-benzylisoquinoline alkaloid, is the main bioactive component of *Stephania tetrandra* and other related members of the *Menispermaceae* family, which is traditionally used in East Asia as a treatment for various diseases [8,9]. CPA exhibits multiple pharmacological activities, including immunomodulatory, anti-inflammatory, antioxidant, anticancer, antiparasitic, and antiviral properties [10,11]. Recent reports have shown that CPA is a potent candidate against COVID-19 as CPA inhibits nuclear factor-kappa B (NF-κB) activation, cytokine production, lipid peroxidation, as well as nitric oxide production, all of which are important to inflammatory responses and viral replication [12,13]. However, a short half-life, poor water solubility, and oral bioavailability limit its pharmacological application [14].

There are several limitations to conventional drug delivery systems, including poor stability, short circulation times, decreased bioavailability, and off-target distribution [1]. Various nanocarriers attached to specific ligands have been proposed to address these shortcomings [15]. Unfortunately, common nanocarriers are rapidly recognized and phagocytosed by the reticuloendothelial system (RES) [16,17]. In addition, the complex physiological environment limits the targeting capability of nanocarriers [18]. As a promising medical frontier, living cell-based drug delivery systems exhibit good biocompatibility, inherent targeting capabilities, and low in vivo immunogenicity [19]. Among living cells, erythrocytes have been widely reported as natural carriers that deliver fluorescent contrast agents, therapeutic polymers, and enzymes as human erythrocytes circulate in the blood for up to 120 days [20,21,22]. In previous reports, nanocarriers anchored to erythrocytes have shown great promise in extending circulation and treating lung diseases such as small cell lung cancer, non-small cell lung cancer, and metastatic lung cancer [23,24]. After intravenous (IV) injection, nanocarriers were scraped from erythrocytes due to the strong shearing stress between the erythrocytes and capillaries. Desorbed nanocarriers mainly accumulate in lung endothelial cells, the first capillary through which erythrocyte-nanocarrier complexes pass [25]. This strategy exhibits an inherent lung-targeted delivery capability, which can increase the concentration of the drug in regional lung tissue and ultimately significantly improve the therapeutic efficacy. In addition, erythrocyte carriers can block formation of the protein corona around the nanocarriers, further preventing the uptake and clearance of nanocarriers by the RES [23].

**Figure 1:**
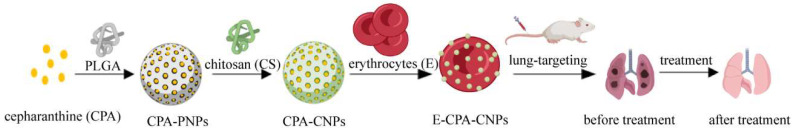
Schematic diagram of an erythrocyte-anchoring strategy. CPA-CNPs were prepared and bound to erythrocytes in a non-covalent manner. After injection into ALI mice, CPA was delivered to the lung to alleviate inflammatory damage.

Chitosan (CS), a natural amino-polysaccharide with high reliability due to its excellent biocompatibility and biodegradability, has been widely used to prepare positively charged nanocarriers [26,27]. With the erythrocyte-anchoring strategy, chitosan nanoparticles loading with doxorubicin or methylprednisolone have been successfully targeted to the lung to treat lung diseases [28,29]. Nanoparticle properties affect the nanoparticle-to-erythrocyte adhesion efficiency and drug delivery efficiency [30]. Although polylactic-coglycolic acid (PLGA) nanoparticles have been intensively studied for various targeted drug delivery systems, PLGA nanoparticles have poor adhesion to erythrocytes due to the negative potential on both the erythrocyte membrane surface and the nanoparticle surface, which may lead to the rapid detachment of nanoparticles from erythrocytes in vivo, thereby being rapidly cleared [18,31]. Here, by encapsulating chitosan on the surface of PLGA nanoparticles, we explored the flexibility of an erythrocyte-anchored strategy for lung-targeted delivery of CPA for the treatment of ALI, providing further reference for CPA to modulate the lung immune response (Figure 1). In this study, CPA-encapsulated PLGA nanoparticles (CPA-PNPs) were used to address the poor water solubility of CPA and improve its bioavailability. To enhance the erythrocyte adhesion efficiency, negatively charged CPA-PNPs were coated with chitosan (CS) (CPA-CNPs) and adhered to erythrocytes (E-CPA-CNPs) in vitro. After IV administration, the in vivo pharmacokinetics (PKs), targeted delivery properties, and therapeutic efficiency of E-CPA-CNPs were systematically studied. Without any ligand modification, E-CPA-CNPs effectively improved the PKs and bioavailability of CPA, significantly promoted the accumulation of CPA in local lung tissue, and ultimately attenuated lung injury in an ALI mouse model.

## 2. Materials and Methods

### 2.1. Materials and Animals

CPA was obtained from Saen Chemical Technology Co., Ltd. (Shanghai, China). PLGA (75:25, 15,000 kDa) was obtained from Xi’an Ruixi Biological Technology Co., Ltd. (Xi’an, China). Pluronic F68 (8600 kDa) was obtained from Harveybio Gene Technology Co., Ltd. (Beijing, China). CS (low viscosity) and Lipopolysaccharide (LPS) were obtained from Sigma-Aldrich (St. Louis, MO, USA). Cyanine 5.5 (Cy5.5), cyanine7.5 (Cy7.5), chlorpromazine (CPZ), and 1,1′-dioctadecyl-3,3,3′,3′-tetramethylindocarbocyanine iodide (DiI) were obtained from Dalian Meilun Biotechnology Co., Ltd. (Dalian, China). The BCA protein assay kit was obtained from Beyotime Biotechnology Co., Ltd. (Nantong, China). The enzyme-linked immunosorbent assay (ELISA) kits were obtained from Solarbio Biotechnology Co., Ltd. (Beijing, China). Human umbilical vein endothelial cells (HUVEC) and RAW 264.7 macrophages were obtained from the IBMS Cell Resource Center (Beijing, China). The cells were cultured in Dulbecco’s modified Eagle’s medium (DMEM) (Gibco, Waltham, MA, USA) supplemented with 10% fetal bovine serum (FBS) (Gibco) and antibiotics (1% penicillin/streptomycin; Gibco). Male Kunming mice (20 ± 2 g) and male Sprague–Dawley (SD) rats (180 ± 20 g) were obtained from Spyfe Biotech (Permit No. SCXK (Jing) 2019–0010; Beijing, China). All animal experiments complied with the code of ethics in research, training, and testing of drugs issued by the Animal Care and Use Ethics Committee in Beijing Institute of Pharmacology and Toxicology.

### 2.2. Preparation of Nanoparticles

The oil/water emulsion method was used to prepare PLGA nanoparticles (PNPs) [32]. 30 mg PLGA and 2 mg CPA were dissolved in 1.5 mL acetone solution as the organic phase. Then it was slowly added dropwise to 10 mL of 1% (*w*/*v*) pluronic F68 solution. The obtained mixture was stirred overnight to obtain CPA-loaded PNPs (CPA-PNPs). After washing three times with double distilled water, chitosan (CS) was added dropwise to CPA-PNPs under continuous stirring for 30 min, and CS-coating to CPA-PNPs were obtained as CPA-CNPs. Probe-labeled nanoparticles (Cy5.5-NPs or Cy7.5-NPs) were prepared by the oil/water emulsion method to visualize the distribution of nanoparticles.

### 2.3. Characterization of Nanoparticles

NPs size (hydrodynamic diameter) and zeta potential of PNPs and CNPs were detected by dynamic light scattering (DLS) (Litesizer 500, Anton Parr, Austria). After diluting 40 times with double distilled water, the morphology of PNPs and CNPs was studied by scanning electron microscopy (SEM) (S-4800, Hitachi, Tokyo, Japan) and transmission electron microscopy (TEM) (H-7650, Hitachi). The concentration of CPA was quantified at 283 nm by high-performance liquid chromatography (HPLC) (1200 series, Agilent Technologies, Savage, MD, USA). The encapsulation efficiency and drug-loading capacity of CPA-PNPs and CPA-CNPs were calculated as follows:

(1) $$\mathrm{Encapsulation}\;\mathrm{efficiency}\;\left(\%\right)=\frac{W_{\mathrm{total}\;\mathrm{CPA}}-W_{\mathrm{free}\;\mathrm{CPA}}}{W_{\mathrm{total}\;\mathrm{CPA}}}\times 100\%$$

(2) $$\mathrm{Drug}-\mathrm{loading}\;\mathrm{capacity}\;\left(\%\right)=\frac{W_{\mathrm{total}\;\mathrm{CPA}}-W_{\mathrm{free}\;\mathrm{CPA}}}{W_{\mathrm{total}\;\mathrm{nanoparticles}}}\times 100\%$$

where $W_{\mathrm{total}\;\mathrm{CPA}}$ represents the total amount of CPA,$\;W_{\mathrm{free}\;\mathrm{CPA}}$ represents the amount of free CPA in the samples, and $W_{\mathrm{total}\;\mathrm{nanoparticles}}$ indicates the amount of total CPA and carriers.

The CPA release profile from CPA-CNPs was analyzed using a dialysis method [12]. 2 mL of CPA or CPA-CNPs were placed in the dialysis bag (Molecular weight cutoff: 8–14 kDa) and immersed in 50 mL phosphate-buffered saline (PBS, 0.01 M, pH 7.4, containing 0.5% tween 80) at 37 °C. Then, a 2-mL sample was collected from the release medium at preset time intervals (0.5, 1, 2, 4, 8, 12, 24, 36, 48, 60, 72, 84, and 96 h), and 2-mL preheated PBS was added. CPA concentrations in the samples were quantified as described above. The percent cumulative release of CPA from CPA-CNPs was calculated.

### 2.4. Adhesion of Nanoparticles to Erythrocytes (E-CNPs) In Vitro

Whole blood obtained from the abdominal aorta (SD rats or Kunming mice) was placed into heparin tubes to prevent clotting. Erythrocytes were separated by centrifugation at 1000× *g* for 10 min and washed 3 times with precooled PBS. Nanoparticles were added to erythrocytes (10% hematocrit) at a ratio of 4 mg nanoparticles per 1 mL erythrocytes and incubated at 37 °C for 30 min. Subsequently, the erythrocyte pellet was washed with PBS to remove unattached nanoparticles. Acetonitrile was added to the erythrocyte samples, and then the erythrocytes-nanoparticles (E-CNPs) interactions were destroyed by sonication. The concentration of CPA in the sample was analyzed by HPLC at 283 nm. The adhesion efficiency of nanoparticles to erythrocytes were calculated as follows:

(3) $$\mathrm{Adhesion}\;\mathrm{efficiency}\;\left(\%\right)=\frac{W_{\mathrm{CPA}\;\mathrm{on}\;\mathrm{erythrocytes}}}{W_{\mathrm{total}\;\mathrm{CPA}}}\times 100\%$$

where $W_{\mathrm{CPA}\;\mathrm{on}\;\mathrm{erythrocytes}}$ represents the amount of CPA on erythrocyte carriers, and $W_{\mathrm{total}\;\mathrm{CPA}}$ indicates the amount of total CPA in the samples.

### 2.5. Characterization of E-CNPs

#### 2.5.1. Morphology of E-CNPs

Erythrocyte samples were dried for SEM visualization by a standard dehydration protocol [28]. Erythrocyte samples were maintained overnight in glutaraldehyde solution. Then, the samples were sequentially transferred to a gradient ethanol solution (30%, 50%, 70%, 80%, 90%, 95%, and 100%) for dehydration. The sample powder was added to the conductive tape and observed by SEM. Besides, Cy5.5-labled CNPs were prepared and adhered to the surface of erythrocytes (E-Cy5.5-CNPs). Colocalization of erythrocytes and nanoparticles was observed by confocal laser scanning microscopy (CLSM) (LSM 880, Zeiss, Jena, Germany).

#### 2.5.2. Shear Responsiveness of E-CNPs

CNPs adhered to erythrocytes may detach from erythrocytes under certain vascular shear environments. The shear responsiveness of E-CNPs was detected by a microfluidic controlled visual rheometer (Fluidicam Rheo, Formulaction, Toulouse, France) [24,30]. E-CNPs were continuously challenged for 15 min at 37 °C under 1 or 5 Pa shear stress. To prevent the re-adhesion of CNPs, 10% serum was put into E-CNPs samples. The shear responsiveness of E-CNPs was expressed as the change in the adhesion efficiency of CNPs to erythrocytes after different shear stress challenge.

### 2.6. Safety Evaluation

#### 2.6.1. Cell Cytotoxicity Evaluation

To evaluate the cytotoxicity of CPA-CNPs, human umbilical vein endothelial cells (HUVEC) (5 × 10^3^ cells/well) were seeded into culture plates in Dulbecco’s modified Eagle’s medium (DMEM) for 24 h. After treatment with different concentrations of CPA-CNPs for another 48 h, 10 μL of Cell Counting Kit-8 (CCK-8) solution was added to cell culture medium. All cells were then incubated for another 2 h. After incubation, the absorbance of each sample plate was analyzed at 450 nm using a multifunctional microplate reader (Tecan, Spark, Austria). HUVEC treated with 15% (*v*/*v*) dimethyl sulfoxide was used as a positive control. The viability of HUVEC treated with PBS (control) was defined as 100%.

#### 2.6.2. Osmotic Fragility

One hundred microliters of E-CPA-CNPs or free erythrocytes were added to 9.9 mL 0.0–0.9% sodium chloride solution (NaCl) to 1% hematocrit and incubated for 30 min at 37 °C. Free erythrocytes (1% hematocrit) incubated for 30 min in double distilled water or 0.9% NaCl at 37 °C were considered as the positive control or negative control for hemolysis, respectively. After incubation, erythrocytes were removed by centrifugation (4 °C, 1000× *g*, 5 min), and the released hemoglobin in the supernatants was analyzed using the multifunctional microplate reader.

#### 2.6.3. Oxidative Fragility

One hundred microliters of E-CPA-CNPs or free erythrocytes were resuspended in 9.9 mL of PBS containing 3 mM hydrogen peroxide (H_2_O_2_). After 24 h of incubation at 37 °C, erythrocyte samples were centrifuged (4 °C, 1000× *g*, 5 min) to remove erythrocytes. The released hemoglobin in the supernatants was analyzed using the multifunctional microplate reader. Free erythrocytes cultured in double distilled water were considered as controls for complete hemolysis.

#### 2.6.4. Hemagglutination Assay

To evaluate the degree of hemagglutination induced by the adhesion of CNPs, a hemagglutination assay was conducted in U-shaped 96-well plates [28]. A known quantity of CNPs was added to fresh erythrocytes (1% hematocrit). After 30 min incubation at 37 °C, 50-μL erythrocyte samples were placed in the U-shaped well and incubated for 1 h. Free erythrocytes were used as controls.

#### 2.6.5. Survival Rate of Erythrocyte Carriers In Vivo

Free erythrocytes or E-CNPs were labeled with 1,1′-dioctadecyl-3,3,3′,3′-tetramethylindocarbocyanine iodide (DiI) and administered to SD rats to evaluate the survival rate of erythrocyte carriers [33]. Briefly, 10 mL DiI solution (10 μg/mL in PBS) was added to 250 µL of packed erythrocytes. After 20 min incubation at 37 °C, DiI-labeled erythrocytes (DiI-erythrocytes) were washed 3 times with PBS. Next, DiI-erythrocytes were incubated with CNPs as described above to obtain DiI-labeled erythrocyte-CNPs (DiI-E-CNPs). DiI-erythrocytes or DiI-E-CNPs were injected intravenously into donor rats (*n* = 3). Then, 0.2 mL of whole blood was obtained at predetermined time intervals. The fluorescence intensity of the erythrocyte samples was detected at 549/565 nm using the multifunctional microplate reader.

#### 2.6.6. In Vivo Safety Evaluation of E-CPA-CNPs

Healthy mice were randomly divided into 4 groups: PBS (control), free CPA, CPA-CNPs, and E-CPA-CNPs (*n* = 3). During the experiment, water and food were freely available. The daily body weights of the mice were detected. On day 7, all experimental mice were euthanized, and the lungs were obtained, washed with PBS, fixed with 4% paraformaldehyde, sectioned, and stained with hematoxylin and eosin (H&E).

### 2.7. Antiphagocytosis Capability of E-CNPs In Vitro

RAW264.7 macrophages (10^5^ cells/well) were seeded into culture plates in DMEM for 24 h. In the treatment group, cells were incubated with Cy5.5-CNPs or E-Cy5.5-CNPs (10 μM) at 37 °C in the dark. In the control group, cells were first incubated with the phagocytosis inhibitor chlorpromazine (CPZ) (10 μg/mL) for 2 h, and then incubated with Cy5.5-CNPs or E-Cy5.5-CNPs under the same conditions, respectively. After 2 h of incubation, cell samples were washed 3 times with PBS and maintained with 4% paraformaldehyde. Fluorescence signals of cell samples were measured by confocal laser scanning microscopy (CLSM) after a final 10 min incubation with Hoechst 33,258.

### 2.8. In Vivo Pharmacokinetics (PK) Studies

The PK profiles of Cy5.5-CNPs and E-Cy5.5-CNPs were measured in healthy SD rats (50 µM/kg, *n* = 3) [12]. After intravenous injection, rats were anesthetized using isoflurane to collect blood samples. 0.4 mL whole blood was harvested at preset time intervals from the retro-orbital sinus. After centrifugation (4 °C, 1000× *g*, 10 min), plasma was obtained for further analysis. The Cy5.5 concentrations in the rat plasma were quantified at 673/692 nm using the standard calibration curve obtained in the experiment.

### 2.9. In Vivo Biodistribution

Healthy mice were randomly divided into 3 groups: PBS, Cy7.5-CNPs, and E-Cy7.5-CNPs (*n* = 3). At 0.5, 2, 4, 6, 8, 12 h, and 16 h, mice were anesthetized, and in vivo bioluminescence image was observed using the IVIS^®^ in vivo system (IVIS^®^ Spectrum, PerkinElmer, Waltham, MA, USA) at 750/820 nm. To determine the local distribution of the formulation, the mice were sacrificed at 6 h and 16 h after injection. The distribution of Cy7.5 in their major organs was observed and examined in vitro. The fluorescence signals of different organs were analyzed with Living Image^®^ software (Caliper, Alameda, CA, USA).

Furthermore, the distribution of E-CNPs in the lungs was visually assessed by CLSM. Cy5.5-CNPs or E-Cy5.5-CNPs were injected to mice via the caudal vein. 12 h after administration, mice were euthanized and their lungs were obtained, washed with PBS, fixed in optimal cutting temperature compound, and sectioned (10 µm). Nuclei were then stained with 4′,6-diamidino-2-phenylindole (DAPI, 358/461 nm). The blue signals of the nuclei and green signals of the CNPs were observed.

### 2.10. In Vivo Therapeutic Efficacy

The ALI mouse model was induced by LPS. The LPS-induced model is the preferred model for ALI due to its easy induction, high reproducibility, and similar pathophysiological characteristics to humans, and is widely used in lung injury-related research [12,34]. Kunming mice in the experimental group were anesthetized with isoflurane. Next, 50-μL LPS was instilled through the trachea (5 mg/kg). After 5 h, 0.2 mL PBS, free CPA, CPA-CNPs, and E-CPA-CNPs were IV injected (1 mg/kg, *n* = 5). 24 h after administration, mice were euthanized, and the bronchoalveolar lavage fluid (BALF) was harvested. The left lung was lavaged three times with pre-cooled PBS (0.5 mL). After centrifugation (1000× *g*, 10 min), the BALF supernatant was aliquoted and stored at −80 °C until subsequent use.

The inflammatory cells in pellet after centrifugation were counted after treatment with erythrocyte lysis buffer. A part of the right lung was maintained with paraformaldehyde for 24 h and stained with H&E. After weighing, the remaining right lung was dried at 60 °C for 72 h to evaluate pulmonary edema. Finally, the levels of total protein, the tumor necrosis factor-alpha (TNF-α), and interleukin 6 (IL-6) in the BALF supernatant were detected with BCA protein assay and ELISA kits, respectively.

### 2.11. Statistical Analysis

Experimental results are expressed as the mean ± standard deviation (SD). Significant differences between different groups were measured using one-way analysis of variance (ANOVA, post-hoc Tukey multiple comparison test). *p* < 0.05 was considered statistically significant.

## 3. Results

### 3.1. Characterization of Nanoparticles

**Figure 2:**
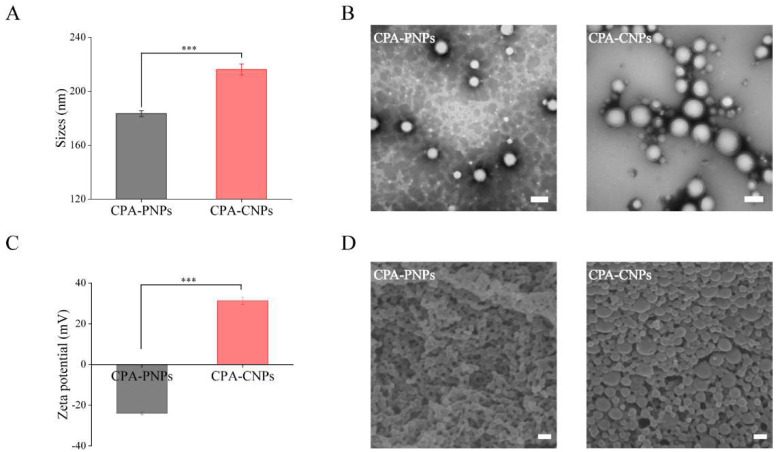
Characterization of nanoparticles. (**A**) The size and (**C**) zeta potential of CPA-PNPs and CPA-CNPs. (**B**) TEM and (**D**) SEM images of CPA-PNPs and CPA-CNPs (scale bar = 100 nm). (*n* = 3, mean ± SD, *** *p* < 0.001).

CPA-PNPs and CPA-CNPs were successfully prepared by oil/water emulsion method, and the mean size and zeta potential of CPA-PNPs and CPA-CNPs were detected by DLS (Figure 2A,C). The mean size and zeta potential of uncoated CPA-PNPs were 183.38 ± 2.21 nm and −23.97 ± 0.63 mV, respectively. After the coating of CS, CPA-CNPs exhibited significant mean size increase (216.09 ± 4.04 nm) and zeta potential transition (+31.30 ± 1.82 mV). The polymer rings (outer shells) and spherical CPA-PNPs (inner cores) could be clearly observed by TEM and SEM (Figure 2B,D). The encapsulation efficiency and drug-loading capacity of the CPA-CNPs were 81.53% and 4.65%, respectively, indicating that the CPA had been efficiently encapsulated in the CNPs. CPA release from CPA-CNPs was determined in a simulated physiological environment. More than 90% of free CPA was released quickly within 1 h, while CPA-CNPs exhibited sustained release behavior within 48 h (Figure S1).

### 3.2. Adhesion of Nanoparticles to Erythrocytes

**Figure 3:**
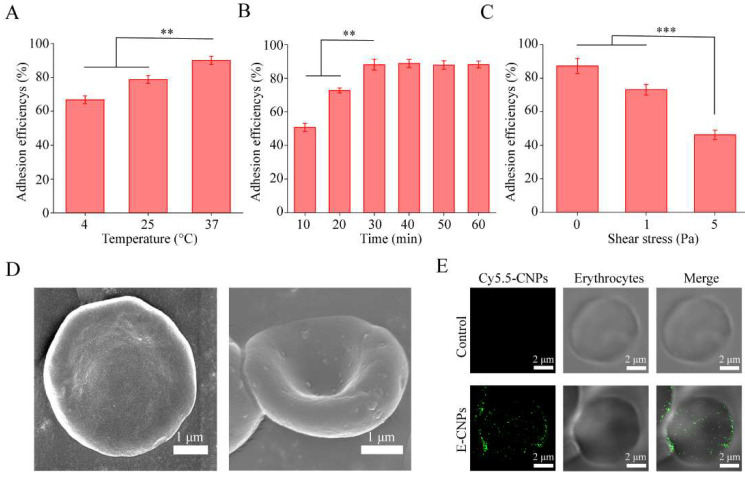
Investigation and characterization of E-CNPs in vitro. The influence of (**A**) incubation temperature and (**B**) incubation time on the adhesion efficiency of CPA-CNPs to erythrocytes. (**C**) CPA-CNPs desorption from erythrocytes under shear stress challenge. Image results of E-CNPs under SEM (**D**) and CLSM (**E**). (*n* = 3, mean ± SD, ** *p* < 0.01, *** *p* < 0.001).

The schematic diagram for E-CNPs is illustrated in Figure 1. CPA-PNPs or CPA-CNPs were adhered to the surface of erythrocytes. The adhesion efficiency of negatively charged PNPs to erythrocytes was about 40%, indicating that PNPs had low affinity to erythrocytes, which may lead to the rapid detachment of CPA-PNPs from the erythrocyte surface and rapid clearance in vivo (Figure S2). Compared with negatively charged CPA-PNPs, the adhesion efficiency of positively charged CPA-CNPs to erythrocytes increased by 113.96% due to the electrostatic interaction between the CPA-CNPs and the erythrocytes. Next, the effects of incubation temperature and time on the adhesion efficiency of CNPs to erythrocytes were further investigated. The adhesion efficiency increased with incubation time and peaked at 30 min (Figure 3A). Similarly, adhesion efficiency increased with incubation temperature, reaching maximum efficiency of 90% at 37 °C and minimum of 30 min (Figure 3B). As a result, the incubation temperature was set to 37 °C and the incubation time was set to 30 min to achieve sufficient adhesion of CNPs to erythrocytes.

### 3.3. Characterization of E-CPA-CNPs

The morphology of E-CPA-CNPs was visualized using SEM. As shown in Figure 3D, the erythrocytes presented as biconcave disks and the spherical CPA-CNPs adhered to the surface of the erythrocytes. The CLSM results showed that Cy5.5-CNPs and erythrocytes were co-localized, illustrating that the E-Cy5.5-CNPs had been successfully prepared (Figure 3E). When subjected to intravascular shear stress, erythrocyte-anchored CNPs may detach from erythrocytes. In normal blood vessels and in pulmonary capillaries, erythrocytes are subjected to 1 Pa shear stress and 5 Pa shear stress, respectively [35]. As shown in Figure 3C, more than 85% of the CPA-CNPs adhered to erythrocytes under static conditions (0 Pa), exhibiting a strong interaction between CPA-CNPs and erythrocytes. Notably, the adhesion was reversible, and the adhesion efficiency dropped to 73% and 46% under 1 Pa and 5 Pa shear stress, respectively. Theoretically, these shear-responsive CPA-CNPs may enable the natural mechanism of nanoparticle transfer.

### 3.4. Safety Evaluation

**Figure 4:**
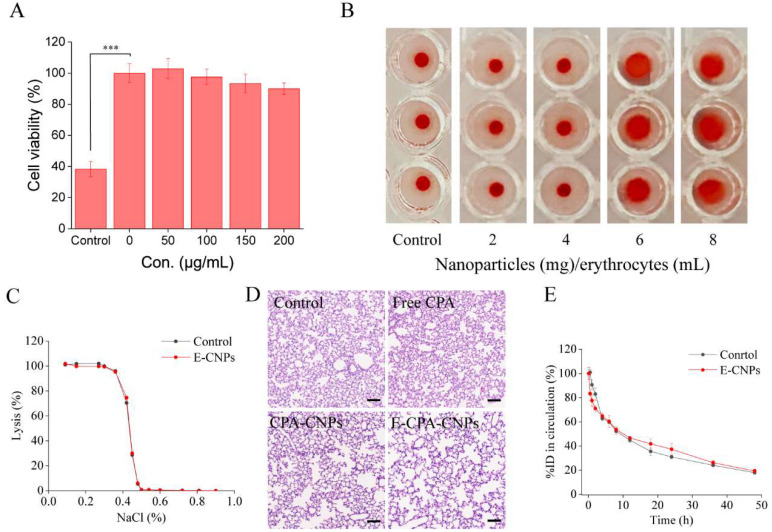
Safety evaluation of E-CNPs. (**A**) Relative cell viability of HUVEC after incubation with different concentrations of CPA-CNPs for 48 h (*n* = 5, mean ± SD). (**B**) Hemagglutination caused by CNPs adhesion in U-shaped 96-well plates (*n* = 3). Unaggregated erythrocytes settle into tight spots at the bottom of the U-plate, while aggregated erythrocytes formed a film over the entire surface of the well. (**C**) Osmotic fragility of free erythrocytes and E-CPA-CNPs (*n* = 3, mean ± SD). (**D**) H&E staining of lung sections from healthy mice injected with various CPA-loaded formulations (scale bar = 100 μm). (**E**) The circulation time of DiI-labeled free erythrocytes and E-CPA-CNPs in vivo. %ID refers to the percentage relative to the injected dose (*n* = 3, mean ± SD, *** *p* < 0.001).

The cytotoxicity of CPA-CNPs in HUVEC was verified using a CCK-8 assay. When the concentration of CPA-CNPs was as high as 200 μg/mL, relative cell viability was still higher than 90%, indicating that the CPA-CNPs had minimal cytotoxicity (Figure 4A). In addition, CNPs adhesion might damage erythrocyte carriers, and rapid spleen removal of damaged erythrocytes could interfere with targeted drug delivery and might even lead to body damage [24,36]. The damage to erythrocyte carriers was systematically evaluated. Osmotic fragility tests were performed to analyze the resistance of E-CPA-CNPs to hypotonic solutions [37]. The level of hemolysis of E-CPA-CNPs was similar to that of free erythrocytes (Figure 4C). For oxidative fragility, the hemolysis level of E-CPA-CNPs did not significantly increase compared with free erythrocytes when subjected to H_2_O_2_ challenge (Figure S3). Hemagglutination tests were performed after adding different concentrations of CNPs to the erythrocytes (Figure 4B). The aggregated erythrocytes formed a film over the entire surface of the well, while healthy erythrocytes settled in a tight spot in the center of the plate. Erythrocyte samples began to agglutinate when the ratio of CPA-CNPs to erythrocytes exceeded 4 mg CNPs per 1 mL erythrocytes. Additionally, the in vivo circulation time of erythrocyte carriers was investigated to further assess erythrocyte carrier damage. The in vivo circulation time of E-CPA-CNPs was not significantly different compared with free erythrocytes (Figure 4E). Furthermore, no significant loss of weight was detected across all treatments (Figure S4). Compared with the control group, no significant histological alterations in lungs were observed after treatment, indicating that E-CPA-CNPs were relatively safe and did not cause obvious erythrocyte agglutination (Figure 4D). Collectively, E-CPA-CNPs displayed great biocompatibility in vitro and in vivo.

### 3.5. Long Circulation Features

**Figure 5:**
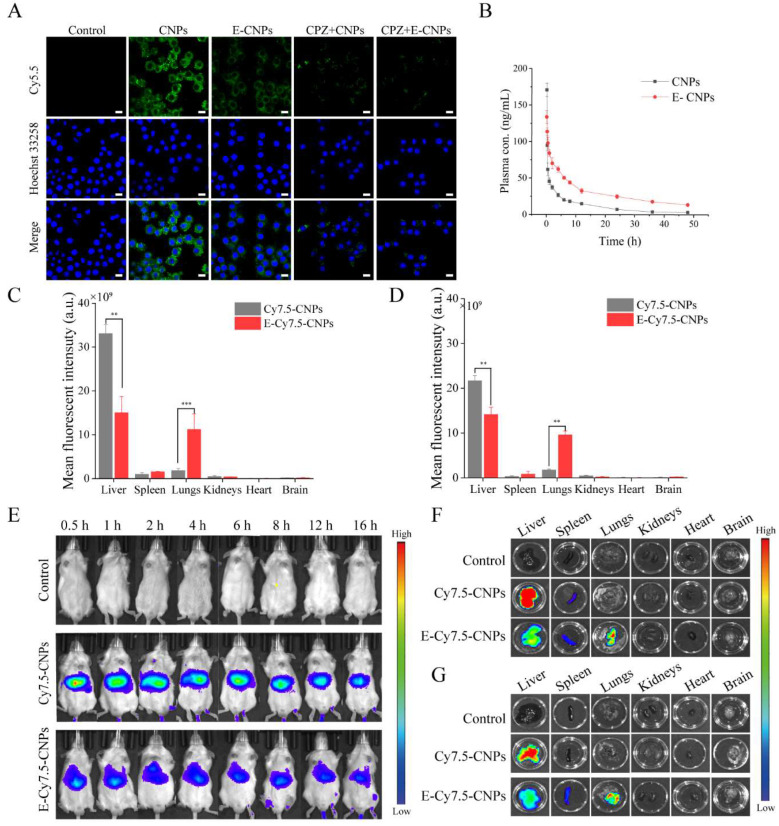
Long circulation features and biodistribution evaluation of E-CNPs in vivo. (**A**) CLSM visualization images of RAW264.7 macrophages incubated with Cy5.5-CNPs or E-Cy5.5-CNPs for 2 h (scale bar = 10 μm). (**B**) Mean plasma Cy5.5 concentration in blood following single-dose IV injection of Cy5.5-CNPs or E-Cy5.5-CNPs in rats (*n* = 3). (**C**–**G**) Distribution of fluorescent signals in mice. Mice were injected intravenously with Cy7.5-CNPs or E-Cy7.5-CNPs and observed at preset time points using a small animal imaging system. The mice were euthanized 6 and 16 h after administration, and their organs were obtained, imaged, and analyzed. Levels of Cy7.5 signal accumulated in the lungs and liver at 6 h (**C**) and 16 h (**D**) after injected. (**E**) Biodistribution analyses of Cy7.5-labeled formulations in vivo. Biodistribution of Cy7.5 fluorescence signal images in isolated organs at 6 h (**F**) and 16 h (**G**). (*n* = 3, mean ± SD, ** *p* < 0.01, and *** *p* < 0.001).

To evaluate whether the erythrocyte-anchoring strategy inhibits the phagocytosis of RES, macrophage uptake of Cy5.5-CNPs or E-Cy5.5-CNPs was analyzed by CLSM after 2 h of co-incubation [38]. In the control group, the intracellular fluorescence intensity of RAW264.7 macrophages was significantly reduced after chlorpromazine treatment, indicating that chlorpromazine could significantly inhibit the phagocytosis of nanocarriers by RAW264.7 macrophages (Figure 5A). The E-CNPs group showed lower fluorescence intensity compared with the bare CNPs group, indicating that E-CNPs significantly reduced the phagocytosis of RAW264.7 macrophages.

The PK studies were further conducted to investigate the long circulation feature of E-CNPs. As shown in Figure 5B, bare CNPs were rapidly cleared from the bloodstream (8.72% retention in circulation after 12 h); however, E-CNPs remained in the bloodstream for extended periods of time (24.30% of E-CNPs retention after 12 h). Half-life, mean residence time (MRT), and the area under the curve (AUC) of E-CNPs were significantly increased compared with bare CNPs (Table S1).

### 3.6. In Vivo Biodistribution

The biodistribution of Cy7.5-CNPs and E-Cy7.5-CNPs in vivo was evaluated to verify the lung-targeted drug delivery capability of E-CNPs. Compared with the E-Cy7.5-CNPs group, the bare Cy7.5-CNPs group showed significant fluorescence signals in the liver due to RES recognition and phagocytosis (Figure 5C–G) [39]. By contrast, in the E-Cy7.5-CNPs group, high fluorescence accumulation was observed in the lung within 0.5 h after administration (Figure 5E). The quantitative fluorescence results of the isolated lung further confirmed that E-CNPs-treated mice showed higher fluorescence intensity. After 6 and 16 h, the lung fluorescence intensity of the E-Cy7.5-CNPs group was enhanced by 6.42-fold and 5.43-fold, respectively, compared with the CNPs group (Figure 5C,D). In contrast, the fluorescence intensity of the liver from the E-CNPs group decreased by 2.2-fold and 1.53-fold after 6 and 16 h, respectively (Figure 5C,D). The green fluorescence signals of CNPs in the lung tissue sections were visualized by CLSM (Figure S5). After 12 h, only a small number of green CNPs was detected in the lung tissue sections of the CNPs group, while a higher intensity of CNPs was detected in the lung tissue sections of the E-CNPs group. Taken together, these results showed that E-CNPs can significantly promote drug delivery and accumulation to the lungs.

### 3.7. In Vivo Therapeutic Efficacy

**Figure 6:**
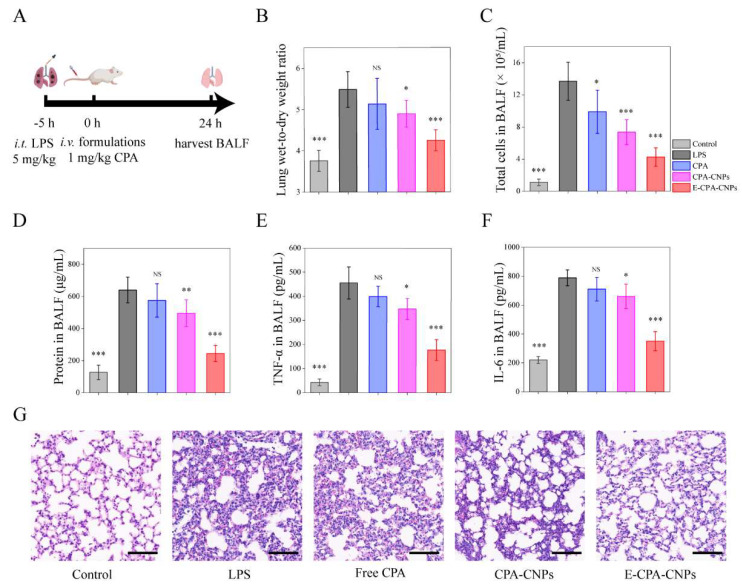
The therapeutic efficiency of E-CPA-CNPs in ALI model mice. (**A**) Schematic illustration for detecting the therapeutic efficiency of free CPA, CPA-CNPs and E-CPA-CNPs (1 mg/kg). (**B**) The lung wet-to-dry weight ratio and (**C**) cell counts in BALF were assessed. (**D**) Measurement of protein content in BALF. The levels of (**E**) TNF-α and (**F**) IL-6 in BALF were detected with ELISA kits. (**G**) H&E staining of lung tissue from each experimental group (scale bar = 50 μm). (*n* = 5, mean ± SD, * *p* < 0.05, ** *p* < 0.01, *** *p* < 0.001 and NS: non-significance, compared with LPS group).

The therapeutic efficacy of E-CPA-CNPs was investigated in an LPS-induced ALI model [12]. At 5 h after LPS challenge, PBS, free CPA, CPA-CNPs, or E-CPA-CNPs were injected (Figure 6A). ALI was characterized by diffuse alveolar injury, production of inflammatory factors (including IL-6 and TNF-α), accumulation of leukocytes, and disruption of alveolar barrier function, leading to hemorrhage, pulmonary edema, and organ failure [40]. After LPS challenge, the lung wet-to-dry ratio, inflammatory cell count, protein concentration, and the levels of inflammatory factors (IL-6 and TNF-α) in the BALF were significantly increased (Figure 6B–F). Treatment with free CPA, CPA-CNPs, or E-CPA-CNPs attenuated the severity of inflammatory injury to varying degrees. Among all CPA treatment groups, the E-CPA-CNPs group exhibited the most significant effect. In particular, the levels of TNF-α and IL-6 in the E-CPA-CNPs group were markedly lower than those in the CPA-CNPs and free CPA groups.

Next, the anti-inflammatory activity of E-CPA-CNPs was determined at the tissue level (Figure 6G). The LPS group exhibited significant bronchial wall thickening, interstitial edema, and inflammatory cell infiltration. CPA formulation administration improved alveolar damage and increased lung injury scores (Figure S6). For the E-CPA-CNPs treatment group, the number of infiltrating inflammatory cells was significantly reduced, and the alveoli were almost intact, indicating that E-CPA-CNPs had significant efficacy in the treatment of ALI.

## 4. Discussion

Our study further demonstrated the feasibility of an erythrocyte anchoring strategy to deliver CS-coating CPA-encapsulated PLGA nanoparticles for the treatment of ALI. Our results showed that coating CS on the surface of PLGA nanoparticles (CNPs) inverted the nanoparticle surface potential to a positive charge, which enabled a strong electrostatic interaction with negatively charged erythrocytes. Therefore, CNPs could tightly bind to erythrocytes and exhibited high erythrocytes adhesion efficiency (Figure 3).

E-CPA-CNPs displayed great biocompatibility (Figure 4). Generally, the adhesion of nanocarriers to erythrocytes may lead to hemolysis or hemagglutination, and the damage to erythrocytes by the preparation of E-CNPs is unavoidable. Our results suggest that erythrocyte damage can be minimized by controlling the concentration of NPs and optimizing the preparation conditions, consistent with previous research [25,28]. RES evasion features are critical for the efficient anti-inflammatory property of CPA-CNPs. E-CPA-CNPs could significantly inhibit the phagocytosis of CPA-CNPs by macrophages, thereby prolonging the in vivo circulation time and improving bioavailability of CPA (Figure 5). This was critical to enhance the in vivo anti-inflammatory efficacy of CPA.

The lung is the first downstream organ after IV injection and receives total cardiac output [41,42]. Shear-responsive E-CPA-CNPs remained intact when exposed to normal vessels (shear stress ~1 Pa) (Figure 3). By contrast, CPA-CNPs were scraped from erythrocytes and accumulated in the lungs during cycling, because they failed to resist crushing and shearing between erythrocytes and capillaries (shear stress ~5 Pa) when E-CPA-CNPs passed through the pulmonary capillaries [35]. Accordingly, E-CPA-CNPs exhibited desirable properties for targeted delivery (Figure 5). Through the erythrocyte-anchoring strategy, E-CNPs can significantly reduce the accumulation of CPA in the liver after intravenous administration and promote the targeted delivery of CPA to the lung (up to 6.42-fold). In theory, E-CNPs can deliver CNPs to any downstream organ of the injection by controlling the adhesion between CNPs and erythrocytes.

To explore the anti-inflammatory effect of CPA in ALI, a mouse model was successfully induced by LPS challenge. The LPS-induced model is the preferred model for ALI due to its easy induction, high reproducibility, and similar pathophysiological characteristics to humans, and is widely used in lung injury-related research [12,34]. CPA is an alkaloid with broad anti-inflammatory activity with various known effects [10]. Delivering CPA to the lung can suppress the inflammatory response and alleviate inflammatory damage (Figure 6). In the E-CPA-CNPs group, CPA-CNPs were targeted to the pulmonary capillary endothelial cells and released CPA consistently. As a result, high concentrations of CPA downregulated the levels of TNF-α and IL-6, thus suppressing a more severe inflammatory response. Collectively, the best anti-inflammatory activity of the E-CPA-CNPs group was mainly attributed to prolonged circulation time and increased CPA concentration in the lungs (Figure 5).

E-CPA-CNPs formulations can safely and effectively target CPA-CNPs to the lungs without any modification of the affinity moiety. Although there is no precise consensus on the optimal clinical dose of CPA, E-CPA-CNP provides an alternative approach to improve CPA PKs and bioavailability and reduce the clinical dose of CPA, which deserves further study. Furthermore, previously reports have suggested that liposomes and nanogels are the best performers for erythrocyte carriers [24,25]. Our results exhibited that optimized CNPs are excellent candidates with a simple preparation process and good biocompatibility. Indeed, nanoparticle properties have a direct impact on the in vivo behavior of erythrocyte-nanoparticle complexes. Future studies are necessary to optimize the nanoparticle properties. Collectively, the E-CPA-CNPs in this study showed great prospect in the treatment of various clinical diseases such as pulmonary hypertension and respiratory infectious diseases (including COVID-19).

## 5. Conclusions

In summary, this study developed an erythrocyte-anchoring strategy for the targeted delivery of CPA to the lungs to treat ALI. By prolonging the circulation time of CNPs, enhancing lung targeting delivery, and increasing CPA accumulation in the lungs, E-CPA-CNPs maximized treatment efficiency while minimizing off-target effects and side effects. Treatment with E-CPA-CNPs offers an effective way to improve CPA PKs and bioavailability, suppress the production of inflammatory cytokines, and ultimately alleviate lung inflammation in ALI disease models.

## Data Availability

All data needed in the paper are present in the paper and in Supplementary Materials. Additional data which are related to this paper may be requested from the authors.

## Supplementary Materials

The following supporting information can be downloaded at: https://www.mdpi.com/article/10.3390/pharmaceutics14091820/s1, Figure S1: In vitro CPA release profile from CPA-CNPs in PBS (0.01 M, pH 7.4, containing 0.5% tween 80) at 37 °C; Figure S2: The adhesion efficiency of NPs to RBCs. Figure S3: Oxidative fragility assay of E-CPA-CNPs. Results were expressed as percent hemolysis of E-CPA-CNPs after being challenged with H_2_O_2_ for 24 h. Figure S4: The change of mouse body weight after administration of PBS (A), free CPA (B), CPA-CNPs (C), or E-CPA-CNPs (D). Figure S5: CLSM visualization of Cy5.5 distribution in the lung at 12 h. Mice were injected intravenously with Cy5.5-CNPs or E-Cy5.5-CNPs. 12 h after injection, mice were euthanized and their lungs were harvested, fixed, and sectioned. Figure S6: Lung injury score of ALI model mice in each CPA formulation group. Table S1: Pharmacokinetic parameters of Cy7.5-CNPs and E-Cy7.5-CNPs.
