# Supplementary material for: Lung-Targeted Delivery of Cepharanthine by an Erythrocyte-Anchoring Strategy for the Treatment of Acute Lung Injury
Source: Pharmaceutics. 2022 Aug 29;14(9):1820. doi: 10.3390/pharmaceutics14091820 (PMC9505324; doi:10.3390/pharmaceutics14091820)
Supplement: Supplementary file 1 [file pharmaceutics-14-01820-s001.zip › pharmaceutics-1823991-supplementary.pdf]

Supplementary Materials:

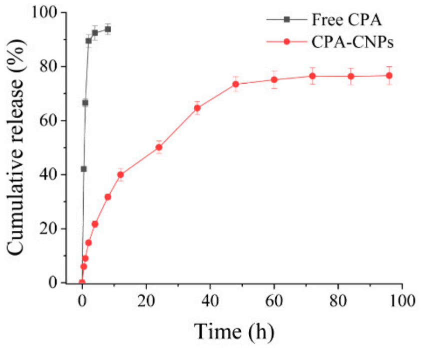

Figure S1. In vitro CPA release profile from CPA-CNPs in PBS (0.01 M, pH 7.4, containing 0.5% tween 80) at 37°C. (n = 3, mean ± SD).

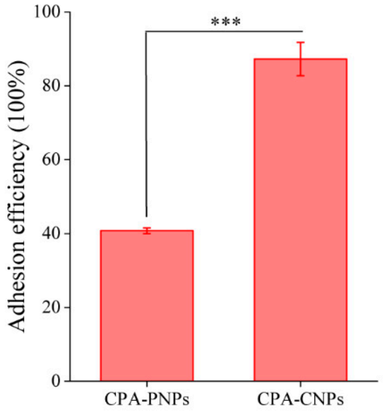

Figure S2. The adhesion efficiency of NPs to RBCs. (n = 3, mean ± SD, *** $p < .001$)

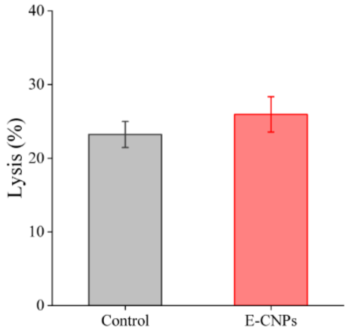

Figure S3. Oxidative fragility assay of E-CPA-CNPs. Results were expressed as

percent hemolysis of E-CPA-CNPs after being challenged with H_2O_2 for 24 h. (n = 3, mean ± SD)

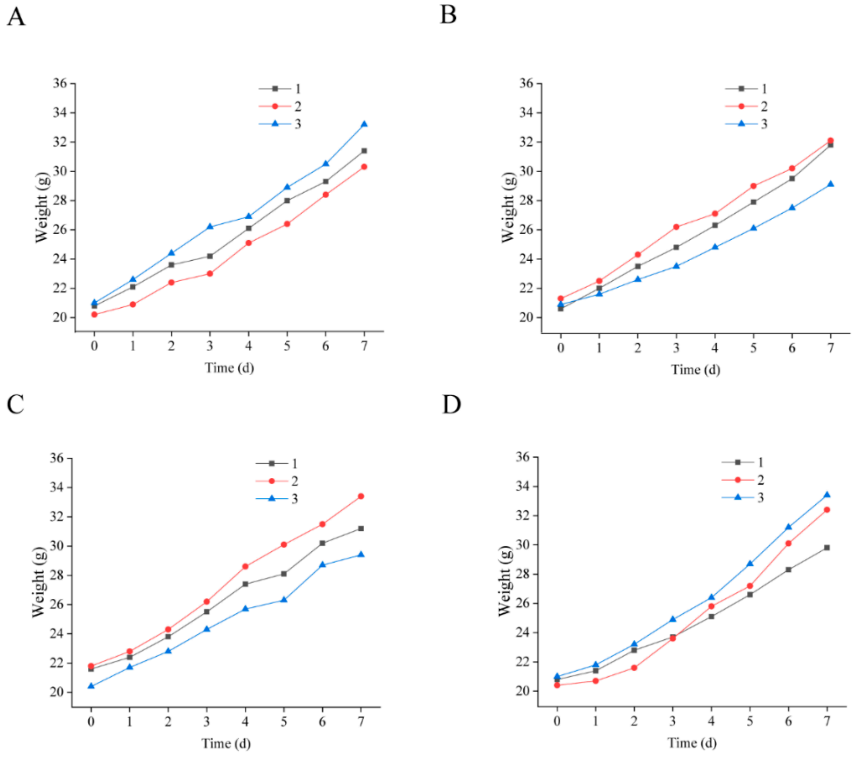

Figure S4. The change of mouse body weight after administration of PBS (A), free CPA (B), CPA-CNPs (C), or E-CPA-CNPs (D). (n = 3)

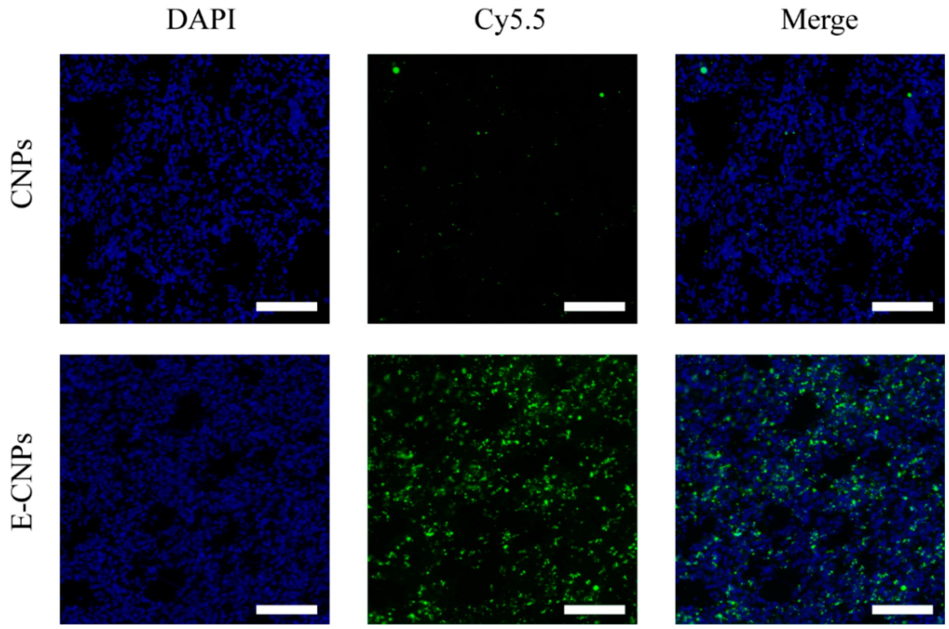

Figure S5. CLSM visualization of Cy5.5 distribution in the lung at 12 h. Mice were

injected intravenously with Cy5.5-CNPs or E-Cy5.5-CNPs. 12 h after injection, mice were euthanized and their lungs were harvested, fixed, and sectioned. ($n = 3$, scale bar = 100 μm).

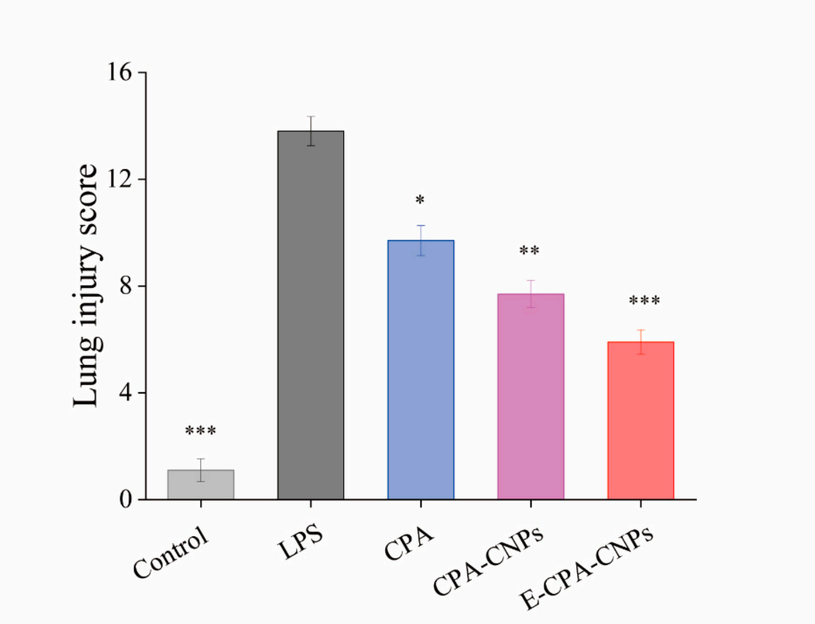

Figure S6. Lung injury score of ALI model mice in each CPA formulation group. The pathology items included lung oedema, alveolar haemorrhage, inflammatory cell infiltration, and alveolar wall thickness. Each item was scored as 0 for normal, and 1, 2, 3, and 4 for mild, moderate, severe, and extremely severe lesions, respectively. The lung injury score is the sum of the scores of each item. Experimental samples were scored by two independent researchers blinded to treatments. ($n = 5$, mean ± SD, * $p < 0.05$, ** $p < 0.01$, *** $p < 0.001$, compared with LPS group)

Table S1. Pharmacokinetic parameters of Cy7.5-CNPs and E-Cy7.5-CNPs. (n = 3).

Parameter	Cy7.5-CNPs	E-Cy7.5-CNPs
AUC_{0-t} (ng/L·h)	417.31 ± 41.86	1053.97 ± 76.16***
$AUC_{0-\infty}$ (ng/L·h)	441.66 ± 54.44	1394.92 ± 142.42***
MRT_{0-t} (h)	12.11 ± 0.62	16.63 ± 0.15***
$MRT_{0-\infty}$ (h)	17.32 ± 1.98	34.04 ± 2.53***
t1/2z (h)	11.84 ± 1.84	25.11 ± 3.33**
CLz (L/h/kg)	183.86 ± 22.17	57.98 ± 6.16**
Vz (L/kg)	3082.31 ± 132.74	2075.96 ± 158.78**
Cmax (ng/mL)	123.39 ± 6.34	96.55 ± 6.21*

$^{*}p < 0.05$, $^{**}p < 0.01$, and $^{***}p < 0.001$ *vs Cy7.5-CNPs*
